# Faster Speciation and Reduced Extinction in the Tropics Contribute to the Mammalian Latitudinal Diversity Gradient

**DOI:** 10.1371/journal.pbio.1001775

**Published:** 2014-01-28

**Authors:** Jonathan Rolland, Fabien L. Condamine, Frederic Jiguet, Hélène Morlon

**Affiliations:** 1 CNRS, UMR 7641 Centre de Mathématiques Appliquées (Ecole Polytechnique), Palaiseau, France; 2 UMR 7204 MNHN–CNRS–UPMC Centre d'Ecologie et de Sciences de la Conservation, Museum National d'Histoire Naturelle, CP51, Paris, France; Australian National University, Australia

## Abstract

The increase in species richness from the poles to the tropics, referred to as the latitudinal diversity gradient, is one of the most ubiquitous biodiversity patterns in the natural world. Although understanding how rates of speciation and extinction vary with latitude is central to explaining this pattern, such analyses have been impeded by the difficulty of estimating diversification rates associated with specific geographic locations. Here, we use a powerful phylogenetic approach and a nearly complete phylogeny of mammals to estimate speciation, extinction, and dispersal rates associated with the tropical and temperate biomes. Overall, speciation rates are higher, and extinction rates lower, in the tropics than in temperate regions. The diversity of the eight most species-rich mammalian orders (covering 92% of all mammals) peaks in the tropics, except that of the Lagomorpha (hares, rabbits, and pikas) reaching a maxima in northern-temperate regions. Latitudinal patterns in diversification rates are strikingly consistent with these diversity patterns, with peaks in species richness associated with low extinction rates (Primates and Lagomorpha), high speciation rates (Diprotodontia, Artiodactyla, and Soricomorpha), or both (Chiroptera and Rodentia). Rates of range expansion were typically higher from the tropics to the temperate regions than in the other direction, supporting the “out of the tropics” hypothesis whereby species originate in the tropics and disperse into higher latitudes. Overall, these results suggest that differences in diversification rates have played a major role in shaping the modern latitudinal diversity gradient in mammals, and illustrate the usefulness of recently developed phylogenetic approaches for understanding this famous yet mysterious pattern.

## Abstract 2

Jonathan Rolland and colleagues show that the gradient of increased mammalian diversity towards the tropics is driven by both faster speciation and reduced extinction.

## Author Summary

Why are there more species in the tropics? This question has fascinated ecologists and evolutionary biologists for decades, generating hundreds of hypotheses, yet basic questions remain: Are rates of speciation higher in the tropics? Are rates of extinction higher in temperate regions? Do the tropics act as a source of diversity for temperate regions? We estimated rates of speciation, extinction, and range expansion associated with mammals living in tropical and temperate regions, using an almost complete mammalian phylogeny. Contrary to what has been suggested before for this class of vertebrates, we found that diversification rates are strikingly consistent with diversity patterns, with latitudinal peaks in species richness being associated with high speciation rates, low extinction rates, or both, depending on the mammalian order (rodents, bats, primates, etc.). We also found evidence for an asymmetry in range expansion, with more expansion “out of” than “into” the tropics. Taken together, these results suggest that tropical regions are not only a reservoir of biodiversity, but also the main place where biodiversity is generated.

## Introduction

The global increase of species richness toward the equator has been the subject of wonder, debates, and speculations since Darwin's times [1],[2]. Why do nearly all groups, spanning from amphibians [3], birds [4],[5], insects [6], mammals [7], and marine invertebrates [8] to micro-organisms [9], have more species in the tropics? Although more than 100 hypotheses have been proposed to explain this latitudinal diversity gradient [10],[11], the number of species in a given clade and region is ultimately explained by four major components: the time since the clade colonized the region, speciation rates, extinction rates, and dispersal events [12]. Hence, three main factors could in principle contribute to the observed high species richness in the tropics: the tropical origin of many clades, higher tropical net diversification rates (speciation minus extinction), and high dispersal rates from temperate regions to the tropics [8],[13].

Two main hypotheses related to dispersal dominate the literature. In the first, known as the “out of the tropics” hypothesis, lineages originate in the tropics, where they massively diversify, and then disperse from the tropics to the temperate regions. Under this hypothesis, dispersal is higher out of than into the tropics, thus acting “against” the latitudinal diversity gradient. In the second hypothesis, known as the “tropical niche conservatism” hypothesis, lineages originate in the tropics and have difficulties to disperse and adapt into temperate regions, thus accumulating in tropical regions [14],[15]. Under both hypotheses, the origin of diversity is tropical, such that intense dispersal from temperate to tropical regions is not considered a plausible explanation for high tropical species richness.

Dispersal effects aside, two major factors remain: time and diversification rates. The relative contribution of these two factors in explaining high tropical species richness remains highly debated [2],[16]. Some hypotheses emphasize diversification rates as the main driving force underlying the latitudinal diversity gradient: the “tropics as cradle” hypothesis emphasizes the role of high tropical speciation rates, whereas the “tropics as museum” hypothesis emphasizes the role of low tropical extinction rates [17]–[20]. Other hypotheses instead emphasize the role of time and historical contingencies [21]. Earth was mostly tropical before temperate regions started to expand ∼30–40 million years (Myr) ago, such that many groups likely have a tropical origin [16], and thus had more time to diversify in the tropics [17],[18].

Several studies, including two recent global-scale phylogenetic analyses of mammals [22] and birds [23], did not detect any correlation between latitude and diversification rates, supporting the view that the latitudinal gradient in species richness is unlinked to differences in diversification rates (e.g., [3],[12]). These findings, however, remain highly debated [24]. For example, Weir and Schluter [25] found a striking effect of latitude on speciation and extinction rates over the last ∼10 Myr in mammals and birds, with an unexpected increase in speciation rates with latitude. A latitudinal gradient in diversification rates has been suggested by several phylogenetic studies of diverse taxa [4],[6],[26],[27], as well as paleontological studies [8],[28],[29], and has given rise to many hypotheses of why speciation and extinction rates may vary with latitude [2]. It has been suggested that speciation is enhanced in the tropics by higher seasonal and longer term climatic stability [30], area effects [31], increased strength of biotic interactions [32],[33], and higher energy [34]. On the other hand, climatic variations and in particular glaciation cycles may be responsible for large-scale extinction events (and potentially enhanced speciation, [25]) in temperate regions [25],[30],[32].

**Figure 1:**
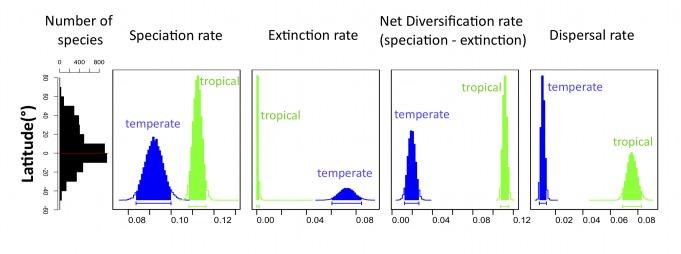
Support for the “out of the tropics” scenario of mammalian species richness. From left to right, global latitudinal diversity gradient of all mammals, and posterior distributions of speciation, extinction, net diversification, and dispersal rates corresponding to the temperate (in blue) and tropical biomes (in green). Faster speciation and reduced extinction in the tropics result in a higher net diversification rate. Range expansion from the tropics to the temperate regions is more frequent than the other way around. Posterior distributions were computed using MCMC analyses for the best-fitting model on the consensus tree. Bars below each distribution correspond to the shaded area and represent the 95% credibility interval of each estimated parameter. Speciation rate refers to within-biome speciation; speciation by biome divergence, which contributes to species richness in the tropical and temperate regions equally, is not included in this figure.

Hence, the relative role of biogeographical history, speciation, and extinction in the latitudinal gradient remains unclear, representing a major scientific challenge for evolutionary biologists [2]. Here, we test the effect of latitude on speciation, extinction, and dispersal rates in the charismatic, species-rich, and globally distributed group of mammals, which displays a striking latitudinal diversity gradient ([7], Figure 1). Studies of the latitudinal gradient in mammals have mainly focused on environmental correlates of species richness [1],[31], and there is currently no consensus as to whether and how diversification rates vary with latitude in this group [2]. Weir and Schluter [25] suggested that both speciation and extinction rates increase with latitude, whereas Soria-Carrasco and Castresana [22] did not find any effect of latitude on speciation, extinction, or net diversification rates. These studies relied on sister taxa [25] or genus-level [22] analyses, thus focusing on recent diversification rates.

Here, we used recently developed biogeographic approaches (GeoSSE, [35]) that allow estimating speciation and extinction rates associated with specific biomes [36],[37]. Similar but nonbiogeographic models [38],[39] have been successfully used to detect various traits affecting diversification rates (e.g., [40]–[42]). Using these recent approaches allowed us to analyze a nearly complete phylogeny of 5,020 mammalian species covering the ∼170 Myr of their evolutionary history [43]–[45].

## Results

### Global Scale, Time-Constant Results

**Figure 2:**
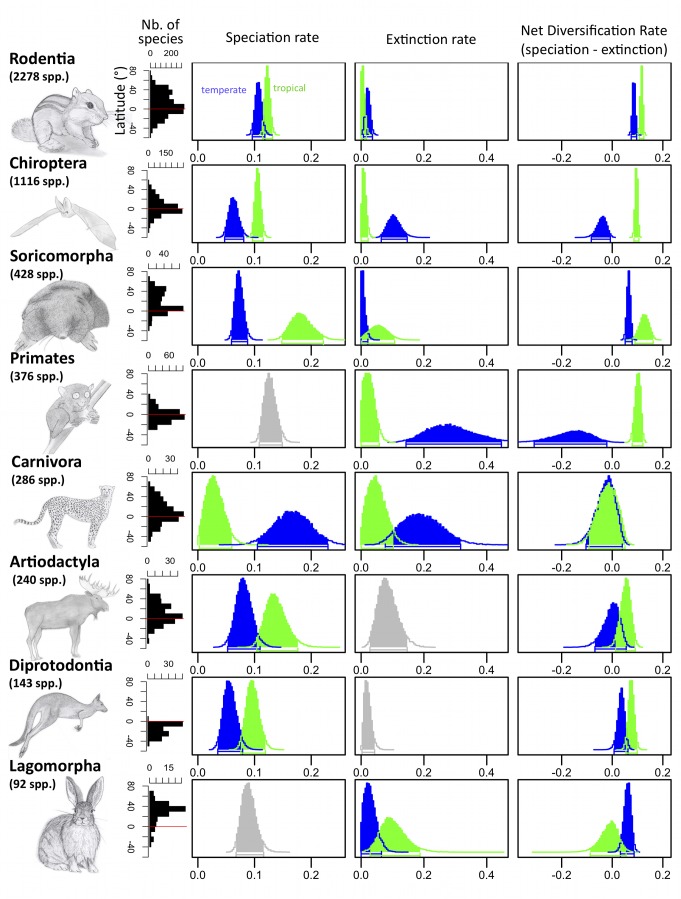
Diversification rates are consistent with diversity patterns across mammalian orders. (Left panels) Mammalian orders (the eight most species-rich orders—covering 92% of all mammals—are represented, ranked from most to least diverse), their total species richness, and their global latitudinal diversity gradient. (Right panels) Posterior distributions of temperate (in blue) and tropical (in green) speciation, extinction, and net diversification rates estimates, computed using the best-fitting model. The grey color indicates that the best-fitting model had equal rates in the tropical and temperate biomes. The net diversification rate follows a trend consistent with the latitudinal diversity gradient: the net diversification rate is higher in the tropics, except in Lagomorpha, which shows an inverse diversity gradient, and in Carnivora, where the difference in net diversification is not significant. Speciation rate refers to within-biome speciation; speciation by biome divergence, which contributes to species richness in the tropical and temperate regions equally, is not included in this figure.

According to current range distribution data from the PanTHERIA database, mammalian species richness peaks near the equator (Figure 1), with 52% of all extant species living in the tropics, whereas only 25% live in temperate regions and 23% span both biomes [46]. The diversity of the eight most species-rich (>75 species) mammalian orders peaks near the equator, except that of the Lagomorpha, which is highest in Northern-temperate regions (Figure 2). We categorized each species reported in the mammalian phylogeny [43]–[45] (Materials and Methods) as living in the tropical biome, the temperate biome, or both. We analyzed the resulting worldwide data using recent biogeographic birth-death models of diversification ([35], Materials and Methods). In these models, a species present in one of the two biomes may give rise to two daughter species in this biome (rate λ), go extinct (rate μ), or disperse and expand its range in the other biome (rate d). These rates of speciation, extinction, and rate expansion may depend (or not) on the species' biome. A species occurring in both biomes (widespread species) may diversify and give rise to either one endemic plus one widespread daughter species (rate λ) or to two endemic daughter species, one in each biome (here referred to as speciation by biome divergence, rate λ_TempTrop_). Speciation by biome divergence can occur if populations belonging to each biome experience directional selection in opposite directions leading to speciation. Widespread species may also contract their range by going extinct in one of the two biomes (rate μ).

We considered 16 alternative diversification scenarios, eight of which included speciation by biome divergence and eight of which did not (i.e., λ_TempTrop_ = 0). Within each of the set of eight scenarios, four had different rates of range expansion from the temperate regions to the tropics than the other way around (d_Temp_≠d_Trop_), and four had equal rates (d_Temp_ = d_Trop_). These four scenarios consisted of (i) a scenario with equal tropical and temperate diversification rates (λ_Temp_ = λ_Trop_ and μ_Temp_ = μ_Trop_), (ii) a scenario with speciation rates differing between biomes but equal extinction rates (λ_Temp_≠λ_Trop_ and μ_Temp_ = μ_Trop_), (iii) a scenario with extinction rates differing between biomes but equal speciation rates (λ_Temp_ = λ_Trop_ and μ_Temp_≠μ_Trop_), and (iv) a scenario with both speciation and extinction rates differing between biomes (λ_Temp_≠λ_Trop_ and μ_Temp_≠μ_Trop_).

We fitted the 16 models to the phylogenetic tree of mammals, accounting for incomplete taxon sampling (Materials and Methods). The best fitting model was the model including speciation by biome divergence and with speciation, extinction, and dispersal rates differing between biomes (Table S1). Estimated speciation rates were higher—and extinction rates lower—in the tropics than in temperate regions (Figure 1, Table S1, and Figure S1), suggesting that the tropics act both as a cradle and as a museum of biodiversity. In addition, estimated rates of range expansion were higher from the tropics to temperate regions than the other way around (Figure 1 and Table S1), supporting Jablonski's “out of the tropics” hypothesis [8],[47]. Analyses on 100 trees randomly sampled from a Bayesian pseudoposterior distribution of trees [44],[45] confirmed these results (Materials and Methods). For all 100 trees, the best-fit diversification model included speciation by biome divergence and suggested the same trends in speciation, extinction, and dispersal rates, with higher speciation rates, lower extinction rates, and higher rates of range expansion in the tropics (Table S2). Estimated rates were consistent with the literature [42],[48]. These results were also robust to an alternative dating of the mammalian phylogeny obtained by incorporating dates from Meredith et al.'s study ([49], Materials and Methods, Table S3).

In our analyses of the global mammalian phylogeny, we made two major simplifying assumptions: that all lineages within a particular biome diversify at the same rate, and that diversification rates remain constant through clades' history. These two assumptions are likely violated in nature: first, diversification rates vary across lineages from a same biome for many reasons, including differences in diets [42], body size [44], or habitats [35],[50]; second, diversification rates typically vary through time [51]. To account for these two sources of rate variation, we carried a series of additional analyses at finer taxonomic resolution (i.e., on smaller phylogenies) and with more complex, time-variable models. For such analyzes, we constrained range expansion to be equally frequent from the tropics to the temperate regions than the other way around. We used this constraint to reach a reasonable trade-off between phylogeny size, model complexity, and statistical power (Materials and Methods). If dispersal rates are higher from the tropics to the temperate regions than the other way around, as estimated from the global phylogeny, constraining these rates to be equal should weaken the biome effect on diversification rather than generating a spurious effect. Trends obtained from dispersal-constrained fits to the whole phylogeny of mammals indeed tended to minimize the effect of latitude on diversification (Figure S2 and Table S4). Thus, further analyses were performed using the eight out of 16 models above with equal rates of range expansion. Comparison of uncertainties around parameter estimates for constrained versus unconstrained models on the global phylogeny suggested that constraining dispersal did not artificially reduce the uncertainty around other parameter estimates (Figures 1 and S2).

### Order and Family Scale, Time-Constant Results

For trees corresponding to the eight richest mammalian orders, the best-fit diversification model varied across groups and trees representing these groups (Table S4). Accounting for speciation by biome divergence improved the fit of the models for the three richest groups (Rodentia, Chiroptera, and Soricomorpha) but not the others, and the estimated rates of speciation by biome divergence were in general lower than within-biome speciation rates. The estimated diversification rates for the richest orders were consistent with estimates obtained from the global phylogeny and in the literature (Figure 2, [42],[48]). There were differences across groups, yet the inferred net diversification rates were consistently higher in the tropics than in temperate regions (Figure 2, Figure S1, and Table S4). The two exceptions concerned the Lagomorpha, for which net diversification rate—following the diversity trend—was higher in temperate regions (Figure 2), and Carnivora, for which tropical and temperate net diversification rates were very similar. The inferred net diversification rate was in general positive except in the temperate regions in Chiroptera and Primates, and in the tropics in Lagomorpha.

Explanations for differences in net diversification rates between biomes differed across orders (Figure 2, Table S4, and Table S5). Higher tropical net diversification rates for Artiodactyla, Diprotodontia, and Soricomorpha were linked to higher speciation rates. In contrast, higher net diversification rates in the tropics for Primates, and in temperate regions for Lagomorpha, were linked to lower extinction rates within their corresponding biomes. Finally, higher tropical net diversification rates resulted from a combined effect of speciation and extinction in Chiroptera and Rodentia. In Carnivora, both speciation and extinction rates were higher in temperate regions, leading to a high species turnover at high latitudes. We tested the robustness of our results to potential biases in the phylogenetic tree we used. We ran our analyses on recent well-sampled phylogenies corresponding to three of the main orders (Rodentia [52], Primates [53], and Carnivora [54]) and the species-rich family *Dasyuridae* within Diprotodontia ([55], Materials and Methods). Even though slightly different models were selected depending on the phylogeny, trends in speciation and extinction rates were highly consistent, suggesting that these trends are strong enough to hold against phylogenetic and dating uncertainties (Table S6). The net diversification trends held in dispersal-constrained analyses as long as range expansion was constrained to not be much more frequent from temperate to tropical regions than in the other direction (Materials and Methods, Table S4). These trends also held when we completely relaxed dispersal, except in Chiroptera, Carnivora, and Lagomorpha, for which dispersal was inferred to contribute significantly to the latitudinal species richness patterns, such that unconstrained models supported different trends in diversification rates than constrained models (Figure S3).

We further refined the taxonomic scale of our analyses by considering all (seven) families with more than 100 species (Table S7). The diversification patterns for families within a given order were generally consistent with the diversification pattern corresponding to that order (Tables S4 and S5): the higher speciation and lower extinction rates in the tropics observed in Chiroptera were also found in its main family Vespertilionidae, the higher tropical speciation and extinction rates observed in Soricomorpha were found in its main family Soricidae, and the lower tropical extinction rate observed in Primates was found in its main family, Cercopithecidae. Higher tropical speciation rates were found in Bovidae, which is the main family of Artiodactyla. In Rodentia, the largest family (Muridae) had higher tropical speciation and extinction rates, consistently with the order. The two other families, however, showed divergent patterns: in Cricetidae the inferred extinction rate was higher in the tropics, and in Sciuridae the inferred speciation rate was higher in temperate regions. Still, net diversification rates were higher in the tropics than in temperate regions for all orders and families, with a single exception for Sciuridae where temperate and tropical net diversification rates were very similar.

### Time-Variable Results

**Figure 3:**
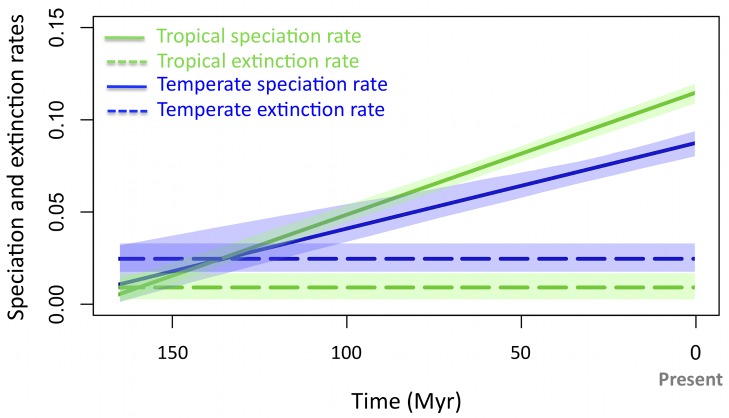
Speciation and extinction rates through time in the temperate and tropical biomes. The speciation rate is higher, and the extinction rate lower, in the tropical biome over the majority of clade history. Lines represent the posterior mean estimates and shaded areas 95% credibility intervals.

Time variation in diversification rates can potentially bias rate estimates [51]. In particular, if speciation rates increased over time in temperate regions—for example, in response to recent glaciations cycles—this could have lead to an accumulation of splitting events towards the tips of the phylogeny. Such increase of splitting events in the recent past would resemble the “pull of the present” effect resulting from extinctions in constant rate models [56] and could thus be spuriously interpreted as a high temperate extinction rate. In order to test the robustness of our results to a potential variation of speciation rates through time, we implemented time variation in the GeoSSE biogeographic model (Materials and Methods). For the global mammalian phylogeny, a model with a linear time dependence of temperate and tropical speciation rates was indeed supported in comparison with the time-constant model (ΔAIC = 93, Table S8). The model suggested an increase in speciation rates through time in both biomes, but this increase did not affect our main findings: the inferred extinction rate remained higher in temperate regions, and speciation rates remained higher in the tropics over the majority of the history of mammals (Figure 3). Estimates obtained for speciation and extinction rates at present were very similar to estimates obtained with the time-constant model (Figure S2, Table S4, and Table S5). The time-variable model was not supported for two of the eight richest orders (Carnivora and Diprotodontia; see AICs in Table S8), suggesting that the hypothesis of time constancy may be relevant at this scale for these orders or that fitting other types of time dependencies would be required to reflect the nonlinear variation of environmental and biogeographic factors that have affected the diversification of mammals across the Cenozoic [48]. The time-variable model revealed trends in time variation (i.e., increase or decline of the speciation rate through time) that varied across orders (Table S8). Despite these variations, the estimated extinction rates remained higher in temperate regions (except in Soricomorpha and Lagomorpha, where they remained lower), and the speciation rates at present remained higher in tropical regions (except in Carnivora, where they remained lower).

## Discussion

The processes underlying the latitudinal diversity gradient are poorly understood. In particular, whether speciation and extinction rates also follow a latitudinal gradient is controversial [16],[22],[23],[25],[26]. The results obtained here for virtually all extant mammal species suggest that both speciation and extinction rates vary strikingly with latitude, resulting in significant differences of net diversification rates between the temperate and tropical biomes. Overall, inferred diversification patterns were consistent with the diversity gradient of each group, with higher speciation rates, lower extinction rates, or a combination of both where diversity is highest. These results were robust to potential variations of diversification rates through time and suggest that differential diversification rates may be largely responsible for today's mammalian diversity patterns.

Our results suggest that speciation rates in mammals are higher in the tropics. This was supported by analyses of the global phylogeny, and in dispersal-constrained analyses for five of the eight orders. This result was also robust to potential variations of diversification rates through time. For Primates and Lagomorpha, no latitudinal difference in speciation was detected, and for Carnivora, a higher speciation rate was found in temperate regions. These results suggest, as proposed by Soria-Carrasco and Castresana [22], that the absence of latitudinal effect on speciation they observed may arise from performing analyses at the genus level, impeding the observation of latitudinal effects that may have occurred in the early history of mammals. Using a phylogeny that covers the entire history of mammalian diversification, we found patterns in line with the hypothesis that speciation rates are higher in the tropics, potentially arising from area effects, increased specialization linked to climatic stability, niche availability, biotic interactions, and higher solar energy [2]. There is a possibility that the latitudinal diversity gradient was shaped during geological times as early as the Late Cretaceous or Paleogene [29] and that differences in diversification rates responsible for the construction of this pattern were only detectable with an approach covering the entire history of mammal diversification [22]. The relevance of this explanation for differences between our results and previous studies is, however, not entirely clear, because a lot of mammalian diversity likely arose in the last ∼20 Myr [57]. In addition, our time-dependent analyses suggest diversification differences between biomes were actually lower early in the history of mammals. Another possibility is that the phylogenetic method we used has more statistical power. In particular, it avoids averaging latitudes across species within genera, which likely weakens the association between latitude and diversification rates.

We found lower extinction rates in tropical regions for the global phylogeny and in dispersal-constrained analyses for the majority of the studied orders (Figures 1–2). A higher extinction rate in temperate regions was already reported for mammals [25] and with fossil records for other taxa [8],[28], potentially arising from higher climatic instability and glaciation cycles in temperate regions [8],[25],[30]. Our estimates of extinction rates in temperate regions were especially high for Primates, most probably due to their distinct preference for tropical forests since they originated in Asia 63–71 Myr ago [53]. Higher extinction rates in tropical regions were only found in Soricomorpha and Lagomorpha. In Lagomorpha, this pattern could come from their hypothesized temperate origin in Mongolia [58], their particularly good adaptation to grasslands (which appeared in the tropics only recently [59]), and strong negative biotic interactions in the tropics (e.g., competition and predation). Similar explanations could explain high extinction rates in Soricomorpha, with some families, such as the Talpidae, originating in temperate regions [60]. The lack of difference between temperate and tropical extinction rates in Diprotodontia may ensue from temperate marsupials having been restricted to low latitudes in the Southern Hemisphere, where they experienced few glaciations, from the extinction of tropical species following the aridification of Australia over the last ∼30 Myr [61], or alternatively from the poor statistical power linked to the paucity of temperate species in this group.

A positive correlation between speciation and extinction rates was found in Carnivora in dispersal-constrained analyses, with higher rates at high rather than low latitudes. This pattern of high turnover was proposed by Weir and Schluter [25] as a general pattern of diversification for recently diverged sister species of birds and mammals, but was observed only for Carnivora in our study. For the other groups, the difference between our results and those of Weir and Schluter [25] may come from differences in spatial scales: we analyzed the global mammalian phylogeny including species from the whole world, whereas Weir and Schluter [25] focused on the New World. In Carnivora, a temperate origin, as suggested in Felidae [62], associated with climatic oscillations and glaciations in temperate regions, may have promoted diversification, either by forcing species to move south or by restricting them to refugia separated by unsuitable habitats [63].

Our results with unconstrained dispersal mostly support Jablonski's “out of the tropics” hypothesis whereby species originate in the tropics and expand their range into temperate regions [8],[47]. This hypothesis was supported by analyses of the global phylogeny and in Rodentia, Soricomorpha, and Artiodactyla. The “out of the tropics” hypothesis has previously found paleontological [8] and phylogenetic [36] support in marine invertebrates, but has to our knowledge not been evidenced for mammals before. In Primates, Chiroptera, Carnivora, and Diprotodontia, range expansion was on the contrary estimated to be more frequent towards the tropics. Accounting for this asymmetry in range expansions did not change diversification trends in Primates and Diprotodontia (although the trend was weakened in the later). In Chiroptera and Carnivora, models with unconstrained dispersal suggested opposite diversification trends, with higher net diversification rates in temperate regions. High range expansion from temperate to tropical regions is not unrealistic. For example, the completion of the isthmus of the Panama 10 to 3 Myr ago resulted in an invasion of the tropics by temperate placental mammals which out-competed tropical marsupials [64]. However, range expansion itself tends to homogenize diversity across latitudes rather than to create a diversity gradient. Thus, high temperate net diversification rates combined with high range expansion from the temperate to the tropical regions cannot explain why there are more species in the tropics in these groups. This suggests that other factors, such as the fact that the tropics are older, are involved. This “time for speciation” hypothesis could be tested in more detail in these groups. More generally, these unconstrained results should be taken with care and tested further given the complexity of the models used relative to the size of the phylogenies [65].

Diversification estimates are only as reliable as the phylogenetic trees used to derive these estimates. Our main analyses were performed on the mammal tree of Bininda-Emonds et al. [43], improved by Fritz et al. [44], and resolved by the polytomy resolver of Kuhn et al. [45]. This tree is the most up-to-date nearly complete species-level tree for mammals, but it has weaknesses in terms of both topology and branch lengths. There is a possibility that the polytomy resolver biased the analyses; however, given that the resolutions are based on a birth-death process with equal rates across the phylogeny, our results of a strong biome effect are conservative. The robustness of the patterns across trees from the posterior distribution is also reassuring in terms of sensitivity to the random resolutions. In addition, diversification patterns in the least resolved groups, Chiroptera and Rodentia, were consistent with patterns obtained in the other groups, which are better resolved. Finally, the consistency of the diversification trends in our analyses of recent, well-sampled phylogenies gives confidence in our results. Another source of uncertainty is the dating of phylogenetic nodes. The family-level tree of Meredith et al. [49] suggests a more ancient origin of mammals and a more recent origin of extant orders (delayed by ∼11 Myr) than the tree of Bininda-Emonds et al. [38], while another family-level tree by dos Reis et al. [66] indicates similar dating results. In agreement with previous results from the literature [49], we found that diversification analyses were consistent between the two alternative dating.

Overall, we advocate that diversification rates play a crucial role in driving differences in species richness between the temperate and tropical biomes. We support the hypothesis that higher mammalian species richness in the tropics results from faster speciation and reduced extinction. This challenges previous suggestions that speciation in mammals is faster in temperate regions [25] or that the latitudinal gradient is linked to factors unrelated to diversification rates, such as the ancient origin of the tropics [2], tropical niche conservatism [16], or higher tropical carrying capacities [67]. Still, further analyses, such as historical biogeographic reconstructions [68] and diversification analyses accounting for nonlinear time dependence [48] and diversity dependence [69],[70], will be needed to fully understand the relative importance of each of these various factors. Mammals are one of the most charismatic and well-documented groups of living organisms, yet our vision of mammalian macroevolution continues to change drastically as new data are compiled and new methods are developed. Similar approaches applied to other vertebrates, insects, plants, and microorganisms will help us meet the challenge of evaluating the roles of history, speciation, and extinction in the origin of the latitudinal diversity gradient. This constitutes a necessary first step before we can fully understand the proximal ecological and evolutionary processes, correlated with latitude, which shaped current diversity patterns.

## Materials and Methods

### Phylogenies

Our main analyses were performed on 100 species-level time-calibrated phylogenetic trees randomly sampled from the Bayesian pseudo-posterior distribution of trees provided by Kuhn et al. [45]. Kuhn et al. [45] used a birth–death model to resolve the polytomies in the supertree of Fritz et al. [44], which was built by completing and redating the phylogeny of Bininda-Emonds et al. [43]. This resulted in 100 phylogenies of 5,020 mammal species each. These phylogenies account for almost all mammals, as the total number of described species is 5,416 [71]. We further used this global-scale distribution of trees to obtain a distribution of trees for each of the eight most species-rich mammalian orders. We also combined these 100 trees to build a maximum clade credibility tree with the TreeAnnotator 1.7.5 (included in the BEAST package [72]). This maximum clade credibility tree, or “consensus” tree, was used for analyses on the whole phylogeny, on the orders and on the families. For convenience, we refer to all these trees as “Bininda-Emonds' trees”.

Dates from Bininda-Emonds' trees are debated [49],[66],[73]. To test the robustness of our results to these dates, we considered a tree incorporating the alternative dating proposed by Meredith et al. [49]. To redate Bininda-Emonds' consensus tree using dates from Meredith et al. [49], we used the list of nodes shared between the two studies given in table 1 from Meredith et al. [49]. Within this list, we selected the 16 deepest nodes, which correspond to ordinal and superordinal groups; these nodes were the ones that diverged the most between the two studies in terms of age estimate. We used these nodes as constraints in PATHd8 [74] to redate Bininda-Emonds' tree (Dataset S1).

Bininda-Emonds' trees offer the unique and considerable advantage to include almost all mammalian species. On the other hand, a significant number of species had no genetic data and were included in the phylogeny by a grafting and random resolution procedure. To test the robustness of our results to this procedure, we selected recent, better sampled phylogenies that did not artificially include species without genetic data. We found four publicly available phylogenies following these criteria that included species from both the tropical and the temperate regions: Rodentia (1155 spp., 50% sampled, [52]), Primates (367 spp., 98% sampled, [53]), Carnivora (286 spp., fully sampled, [54]), and Dasyuridae (Diprotodontia, 66 spp., 96% sampled, [55]).

### Biome Categorization

We obtained minimum and maximum latitudinal data from the PanTHERIA database [46]. These data cover 4,668 species, including 4,536 of the species from the phylogeny. We chose −23.4° and 23.4° as the threshold latitudes defining the tropics and used the latitudinal data to characterize each species as living in the temperate biome, the tropical biome, or both (“widespread” species). We discarded all species in the phylogeny for which latitudinal data were not available, including all marine mammal species.

The GeoSSE model can account for missing species in phylogenies. Likelihoods corresponding to incomplete phylogenies are obtained by considering the probabilities that an extant species from each type (here tropical, temperate, and widespread) is sampled. In practice, these probabilities were computed as the fraction of species from each type present in the phylogeny, and directly introduced as a fixed parameter in the likelihood function. We estimated the fraction of species sampled in each biogeographic category (temperate, tropical, and widespread). We estimated the total number of species in each category by applying the fraction of temperate, tropical, and widespread species computed from the 4,668 species represented in PanTHERIA to the total number of described mammal species (5,416 species following [71]). In Bininda-Emonds' tree, for example, the estimated fraction of species represented in the phylogeny was 0.84 for temperate species, 0.83 for tropical species, and 0.85 for species spanning both biomes.

### Diversification Analyses

To test for an association between latitude and diversification rates, we used the Geographic State Speciation and Extinction model (GeoSSE, [35]), implemented in the *diversitree* R-package [75]. This birth–death model is a geographic extension of character-dependent diversification models [38],[39] including three parameters related to speciation (λ_Temp_, λ_Trop_, λ_TempTrop_), two parameters related to extinction and range contraction (μ_Temp_, μ_Trop_), and two parameters related to range expansion (d_Temp_, d_Trop_). We used two types of analyses: analyses in which the seven parameters were allowed to vary freely, and analyses with constrained dispersal (i.e., d_Temp_ = d_Trop_).

Analyses of the global phylogeny were performed on both the consensus tree and the posterior distribution of trees. We compared the 16 diversification models described in the text using the Akaike Information Criterion (AIC). We checked support for the selected model against all other models nested within it using the likelihood ratio test (*p*<0.05). We estimated the speciation, extinction, and range expansion rates corresponding to the best fitting model. We ran Bayesian Markov chain Monte Carlo (MCMC) analyses on the consensus tree, using exponential priors with parameters obtained from the character independent model, a 500-step burnin, and 20,000-step chain [75]. Convergence occurred within the few first steps and parameter estimates were very stable along the chain (Figure S4).

In our analyses of the eight most species-rich orders of mammals, the main families within these orders, and the four more recent phylogenies described above, we reduced the number of parameters in our model by constraining dispersal (i.e., fixing d_Temp_ = d_Trop_). It is now well recognized that phylogenies have limited statistical power, particularly those of small size [76],[77]. Fitting complex models to such phylogenies is not recommended. In particular, a study specifically designed to test the robustness of character-dependent models—such as the GeoSSE model used here—recommended to simplify models by reducing their number of parameters when phylogenies are small [65]. Given our primary interest to analyze the effect of biomes on speciation and extinction rates, we constrained dispersal. We performed the same maximum likelihood and MCMC analyses as on the global phylogeny, using the eight models with constrained dispersal.

We tested the robustness of our results to the hypothesis that range expansion is symmetric (d_Temp_/d_Trop_ = 1). First, we ran the best-fitting models while constraining the ratio d_Temp_/d_Trop_ to other values, using the consensus tree used for MCMC analyses. We considered 150 values ranging from 0 to +∞, thus encompassing scenarios in which range expansion from the tropics to temperate regions is more frequent (d_Temp_/d_Trop_<1) and scenarios in which range expansion from temperate regions to the tropics is more frequent (d_Temp_/d_Trop_>1). For each value of d_Temp_/d_Trop_, we assessed whether the trend in net diversification rate was conserved. For example, if we found a higher net diversification rate in the tropics under the initial hypothesis of symmetrical range expansion (d_Temp_/d_Trop_ = 1), we assessed if it remained higher in the tropics with the new ratio. This yielded a lowest and highest ratio d_Temp_/d_Trop_ such that trends were conserved. Second, we ran unconstrained models, such as the ones fitted to the global mammalian phylogeny (described above).

### Robustness of the Results to Time Variation in Speciation Rates

To test the robustness of our results to time variation in speciation rates, we relaxed the hypothesis of rate constancy in the GeoSSE model. We modified the make.geosse function from the *diversitree* package [70], which computes likelihood functions associated with the different biogeographic models, by integrating the implementation of time dependency available in the Binary State Speciation and Extinction (BiSSE) model (codes are available in *diversitree*, make.geosse.t function). Speciation rates were assumed to vary linearly through time, such that λ(t) = λ_0_+rt, where λ_0_ is the speciation rate at present and r controls the rate of change in speciation rate through time, and t measures time from the present to the past. Extinction rates and dispersal are assumed constant through time μ(t) = μ, d(t) = d. We included time variation in speciation rates in the best-fit time-constant model (i.e., the model reported in Tables S4 and S5), with d_Temp_ = d_Trop_. These analyses were performed on the consensus “Bininda-Emonds” tree for the global phylogeny and each order.

## Supporting Information

Dataset S1 **Bininda-Emonds et al.'s** [**43**] **tree redated using dates from Meredith et al.** [**49**] **.**
(TXT)

Figure S1 **Differences between tropical and temperate net diversification rates, computed from the MCMC analyses.** Global phylogeny: results from the dispersal-unconstrained model. Order-level phylogenies: results from the dispersal-constrained model. The *x*-axis represents the difference between tropical and temperate net diversification rates (r_Trop_–r_Temp_). The *y*-axis represents the posterior density probability. Grey bars (bottom) correspond to the shaded area and represent the 95% credibility interval of the parameter estimates. The difference is significant if the credibility interval does not encompass 0—that is, for all phylogenies except the Carnivora.
(TIF)

Figure S2 **Analyses of the global mammal phylogeny with constrained dispersal did not artificially reinforce diversification trends nor reduce uncertainties around parameter estimates.** From left to right, global latitudinal diversity gradient of all mammals, and posterior distributions of speciation, extinction, and net diversification rates for temperate (in blue) and tropical biomes (in green), computed using MCMC analyses for the best-fitting model on the consensus tree. Bars below each distribution correspond to the shaded area and represent the 95% credibility interval of the estimated parameter. Speciation rate refers to within-biome speciation; speciation by biome divergence, which contributes to species richness in the tropical and temperate regions equally, is not included in this figure. Constraining dispersal does not artificially reinforce diversification trends: the difference between temperate and tropical net diversification rates is about twice higher when dispersal is unconstrained (9.2×10^−2^ Myr^−1^, Figure 1) than when dispersal is constrained (4.7×10^−2^ Myr^−1^). Constraining dispersal does not artificially reduce uncertainties around parameter estimates. Standard deviation around parameter estimates from constrained and unconstrained MCMC analyses are as follows: λ_Temp_, 3×10^−3^ Myr^−1^ in constrained analyses versus 4×10^−3^ Myr^−1^ in unconstrained analyses; λ_Trop_, 2×10^−3^ Myr^−1^ versus 2×10^−3^ Myr^−1^; λ_TempTrop_, 2×10^−3^ Myr^−1^ versus 2×10^−3^ Myr^−1^; μ_Temp_, 3×10^−3^ Myr^−1^ versus 6×10^−3^ Myr^−1^; μ_Trop_, 4×10^−3^ Myr^−1^ versus 7×10^−4^ Myr^−1^, d, 1×10^−3^ Myr^−1^ versus 1×10^−3^ Myr^−1^.
(TIF)

Figure S3 **MCMC analyses corresponding to diversification models with unconstrained dispersal for the eight richest orders.** (Left panels) Mammalian orders (the eight most species-rich orders—covering 92% of all mammals—are represented, ranked from most to least diverse), their total species richness, and their global latitudinal diversity gradient. (Right panels) Posterior distributions of temperate (in blue) and tropical (in green) speciation, extinction, net diversification, and dispersal rate estimates, computed using the best-fitting model. The grey color indicates that the best-fitting model had equal rates in the tropical and temperate biomes. Speciation rate refers to within-biome speciation; speciation by biome divergence, which contributes to species richness in the tropical and temperate regions equally, is not included in this figure. For five orders (Rodentia, Soricomorpha, Primates, Artiodactyla, and Diprotodontia), the dispersal rate estimates results in ratios of d_Temp_/d_Trop_ that fall into the domain of robustness identified with the constrained models (Table S4). For these groups, trends in net diversification rates found with the unconstrained analyses are in line with the results found with the constrained models: higher net diversification rates are found in the tropics; in Diprotodontia, the trend is conserved but is no longer statistically significant. In the three remaining orders (Chiroptera, Carnivora, and Lagomorpha), dispersal rate estimates result in ratios of d_Temp_/d_Trop_ that fall outside the domain of robustness identified with the constrained models (Table S4). Range expansion is estimated to be very high toward the species-rich region and may contribute substantially to the latitudinal gradient. Net diversification rates become higher in temperate regions in Chiroptera and Carnivora, and higher in tropical regions in Lagomorpha.
(TIF)

Figure S4 **Stability of speciation, extinction, and dispersal estimates along the MCMC.** Estimates of speciation and extinction rates in the temperate and tropical biomes along the 20,000 steps of the MCMC following the 500 steps of burnin (not shown in the figure). Parameter estimates are stable along the chain.
(TIF)

Table S1 **Comparison of models for the global, consensus phylogeny.** The following table reports results corresponding to the 16 models considered in the article, ranked from best (top) to worst (bottom) fit. Numbers report parameter estimates on the consensus tree.
(TIF)

Table S2 **Comparison of models for the global phylogeny, using the 100-tree posterior distribution.** The following table reports results corresponding to the 16 models considered in the article, ranked from best (top) to worst (bottom) fit. Numbers report parameter and standard deviation estimates from a posterior distribution of 100 trees. Uncertainties between the 100 trees from the posterior distribution and within trees, given by the MCMC analyses for one tree (e.g., Figure 1), were of the same order of magnitude of ∼1×10^−3^ Myr^−1^ for both unconstrained and constrained analyses.
(TIF)

Table S3 **Comparison of models for Bininda-Emonds et al.'s** [**43**] **tree redated according to Meredith et al.** [**49**] **.** Results for the eight models considered in the article, ranked from best (top) to worst (bottom) fit. The best-fit model is the model with higher speciation, lower extinction, and higher dispersal rates in the tropics, in agreement with results obtained with the alternative dating. Diversification rate estimates are very similar.
(TIF)

Table S4 **Model selection, parameter estimates, and robustness of the results.** The second column indicates the number of trees, out of 100, for which the dispersal-constraint model specified in the corresponding row is the best. Models supported by less than 10 trees are not shown. Cells filled with parameter estimates define the model; for example, if parameter estimates are specified in the column headed “λ_Temp_ = λ_Trop_,” this indicates that the best-fitting model is a model with equal tropical and temperate speciation rates. The middle columns report mean ± sd parameter estimates over the 100 trees. The two last columns display min and max values of the ratio d_Temp_/d_Trop_ for which the trend in net diversification rates is conserved. Results were robust to the assumption that range expansion is as frequent from the tropics to the temperate region than in the other direction (d_Temp_ = d_Trop_). Estimated net diversification rate remains higher in the tropics than in temperate regions for all groups (except the Lagomorpha for which it remained lower) when range expansion is assumed to be less frequent from the temperate to the tropical regions than in the other direction (d_Temp_/d_Trop_<1), or when range expansion is reasonably more frequent from the temperate to the tropical regions than in the other direction: 1<d_Temp_/d_Trop_<2.7 for the whole phylogeny and 1<d_Temp_/d_Trop_<1.8 for orders other than Carnivora.
(TIF)

Table S5 **Comparison of models across mammalian orders.** The following table reports results corresponding to the eight dispersal-constrained models considered in the article, ranked from best (top) to worst (bottom) fit. Numbers report parameter estimates on the consensus tree built from a posterior distribution of 100 trees.
(TIF)

Table S6 **Comparison of models for the four well-sampled phylogenies from the recent literature.** The following tables report results corresponding to the eight models considered in the article, ranked from best (top) to worst (bottom) fit. In Rodentia [52], the first best model supports higher speciation rates in the tropics, and the second best model supports higher speciation and lower extinction in the tropics, in agreement with results from Bininda-Emonds' trees. In Primates [53], the first best model supports lower extinction rates in the tropics, in agreement with results from Bininda-Emonds' trees, but also higher speciation rates in the tropics. In Carnivora [54], the best model supports higher speciation and extinction rates in the tropics, in agreement with results from Bininda-Emonds' trees. In Dasyuridae (Diprotodontia) [55], the second best model suggests higher speciation rates in the tropics, in agreement with results found in Diprotodontia with Bininda-Emonds' trees. The first model did not detect significant differences between biomes, which could be due to decreased statistical power linked to the small size of the group.
(TIF)

Table S7 **Comparison of models across mammalian families.** The following table reports results corresponding to the eight dispersal-constrained models considered in the article, ranked from best (top) to worst (bottom) fit. The best-fit model for the corresponding order (as reported in Figure 2 and Table S5) was often ranked first; when it was ranked second, it did not differ from the best model by more than 2 AIC values. Trends in diversification rates were generally consistent with trends observed at the order level.
(TIF)

Table S8 **Parameters related to speciation, extinction, and range expansion under the time-varying GeoSSE model.** Speciation rates are assumed to vary linearly through time, such that λ(t) = λ_0_+rt, where λ_0_ is the speciation rate at present, r controls the rate of change in speciation rate through time, and t measures time from the present to the past. Extinction and dispersal rates are assumed constant through time (i.e., μ(t) = μ, d(t) = d). Estimated speciation and extinction rates remain positive over the history of the groups. Extinction rates are higher in the temperate biome except for Lagomorpha and Soricomorpha, and speciation rates at present are higher in the tropics except for Carnivora, in agreement with results found with time-constant models. Time-constant models (shown in grey) are better supported than time-variable models in Carnivora and Dripotodontia.
(TIF)

## Abbreviations

Myr: million years
