# Supplementary figures and images for: Faster Speciation and Reduced Extinction in the Tropics Contribute to the Mammalian Latitudinal Diversity Gradient
Source: PLoS Biol. 2014 Jan 28;12(1):e1001775. doi: 10.1371/journal.pbio.1001775 (PMC3904837; doi:10.1371/journal.pbio.1001775)

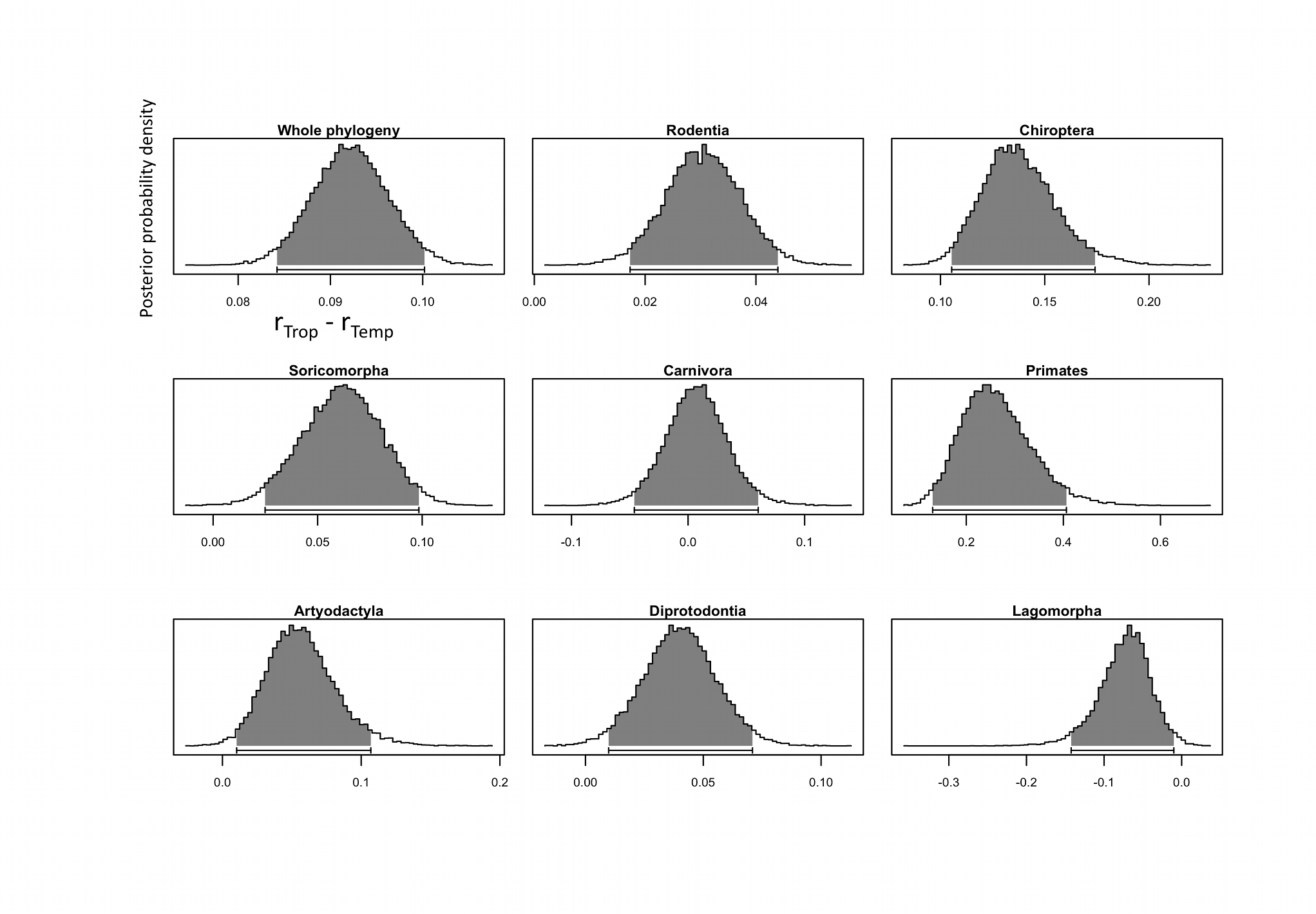

Supplement: Figure S1 — Differences between tropical and temperate net diversification rates, computed from the MCMC analyses. Global phylogeny: results from the dispersal-unconstrained model. Order-level phylogenies: results from the dispersal-constrained model. The x-axis represents the difference between tropical and temperate net diversification rates (rTrop–rTemp). The y-axis represents the posterior density probability. Grey bars (bottom) correspond to the shaded area and represent the 95% credibility interval of the parameter estimates. The difference is significant if the credibility interval does not encompass 0—that is, for all phylogenies except the Carnivora. (TIF) [file pbio.1001775.s002.tif]

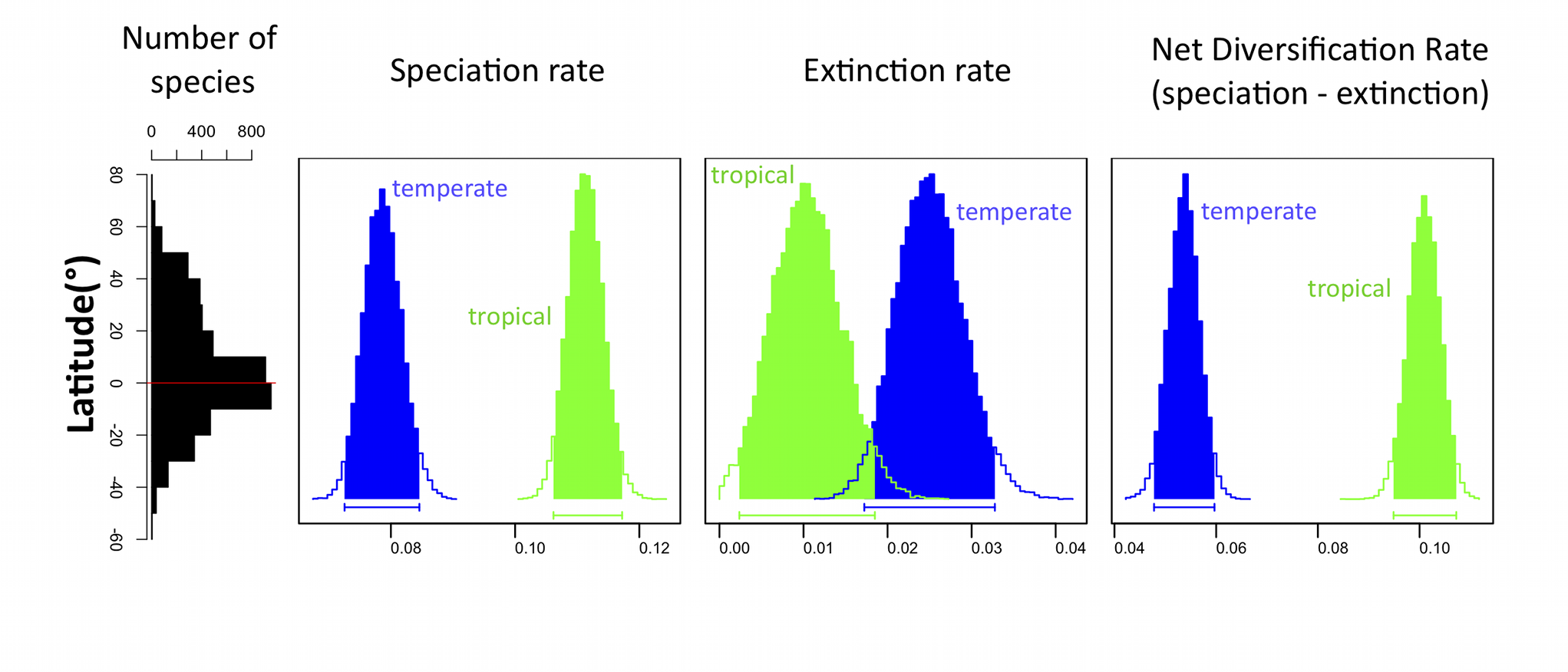

Supplement: Figure S2 — Analyses of the global mammal phylogeny with constrained dispersal did not artificially reinforce diversification trends nor reduce uncertainties around parameter estimates. From left to right, global latitudinal diversity gradient of all mammals, and posterior distributions of speciation, extinction, and net diversification rates for temperate (in blue) and tropical biomes (in green), computed using MCMC analyses for the best-fitting model on the consensus tree. Bars below each distribution correspond to the shaded area and represent the 95% credibility interval of the estimated parameter. Speciation rate refers to within-biome speciation; speciation by biome divergence, which contributes to species richness in the tropical and temperate regions equally, is not included in this figure. Constraining dispersal does not artificially reinforce diversification trends: the difference between temperate and tropical net diversification rates is about twice higher when dispersal is unconstrained (9.2×10^−2^ Myr−1, Figure 1) than when dispersal is constrained (4.7×10^−2^ Myr−1). Constraining dispersal does not artificially reduce uncertainties around parameter estimates. Standard deviation around parameter estimates from constrained and unconstrained MCMC analyses are as follows: λTemp, 3×10^−3^ Myr−1 in constrained analyses versus 4×10^−3^ Myr−1 in unconstrained analyses; λTrop, 2×10^−3^ Myr−1 versus 2×10^−3^ Myr−1; λTempTrop, 2×10^−3^ Myr−1 versus 2×10^−3^ Myr−1; μTemp, 3×10^−3^ Myr−1 versus 6×10^−3^ Myr−1; μTrop, 4×10^−3^ Myr−1 versus 7×10^−4^ Myr−1, d, 1×10^−3^ Myr−1 versus 1×10^−3^ Myr−1. (TIF) [file pbio.1001775.s003.tif]

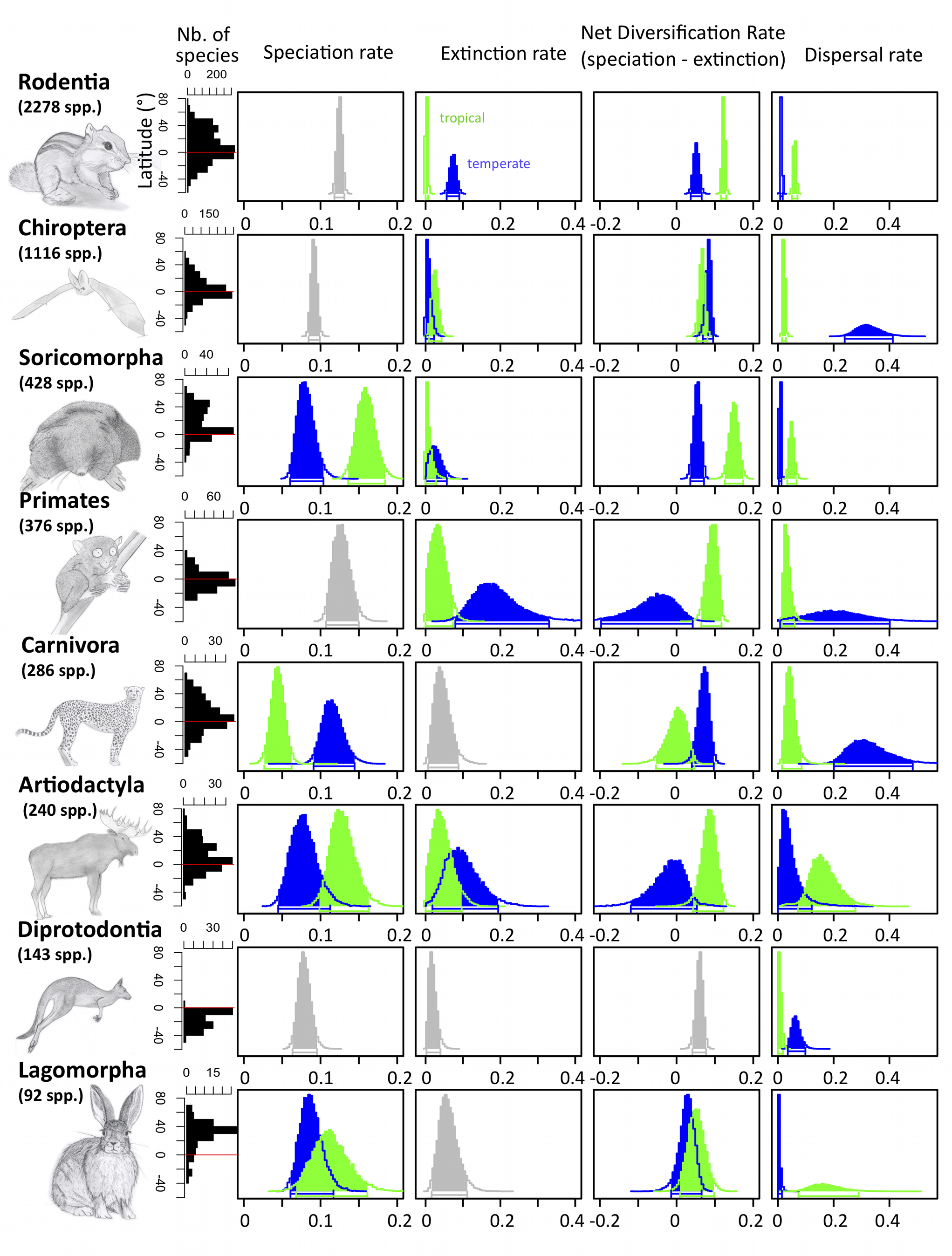

Supplement: Figure S3 — MCMC analyses corresponding to diversification models with unconstrained dispersal for the eight richest orders. (Left panels) Mammalian orders (the eight most species-rich orders—covering 92% of all mammals—are represented, ranked from most to least diverse), their total species richness, and their global latitudinal diversity gradient. (Right panels) Posterior distributions of temperate (in blue) and tropical (in green) speciation, extinction, net diversification, and dispersal rate estimates, computed using the best-fitting model. The grey color indicates that the best-fitting model had equal rates in the tropical and temperate biomes. Speciation rate refers to within-biome speciation; speciation by biome divergence, which contributes to species richness in the tropical and temperate regions equally, is not included in this figure. For five orders (Rodentia, Soricomorpha, Primates, Artiodactyla, and Diprotodontia), the dispersal rate estimates results in ratios of dTemp/dTrop that fall into the domain of robustness identified with the constrained models (Table S4). For these groups, trends in net diversification rates found with the unconstrained analyses are in line with the results found with the constrained models: higher net diversification rates are found in the tropics; in Diprotodontia, the trend is conserved but is no longer statistically significant. In the three remaining orders (Chiroptera, Carnivora, and Lagomorpha), dispersal rate estimates result in ratios of dTemp/dTrop that fall outside the domain of robustness identified with the constrained models (Table S4). Range expansion is estimated to be very high toward the species-rich region and may contribute substantially to the latitudinal gradient. Net diversification rates become higher in temperate regions in Chiroptera and Carnivora, and higher in tropical regions in Lagomorpha. (TIF) [file pbio.1001775.s004.tif]

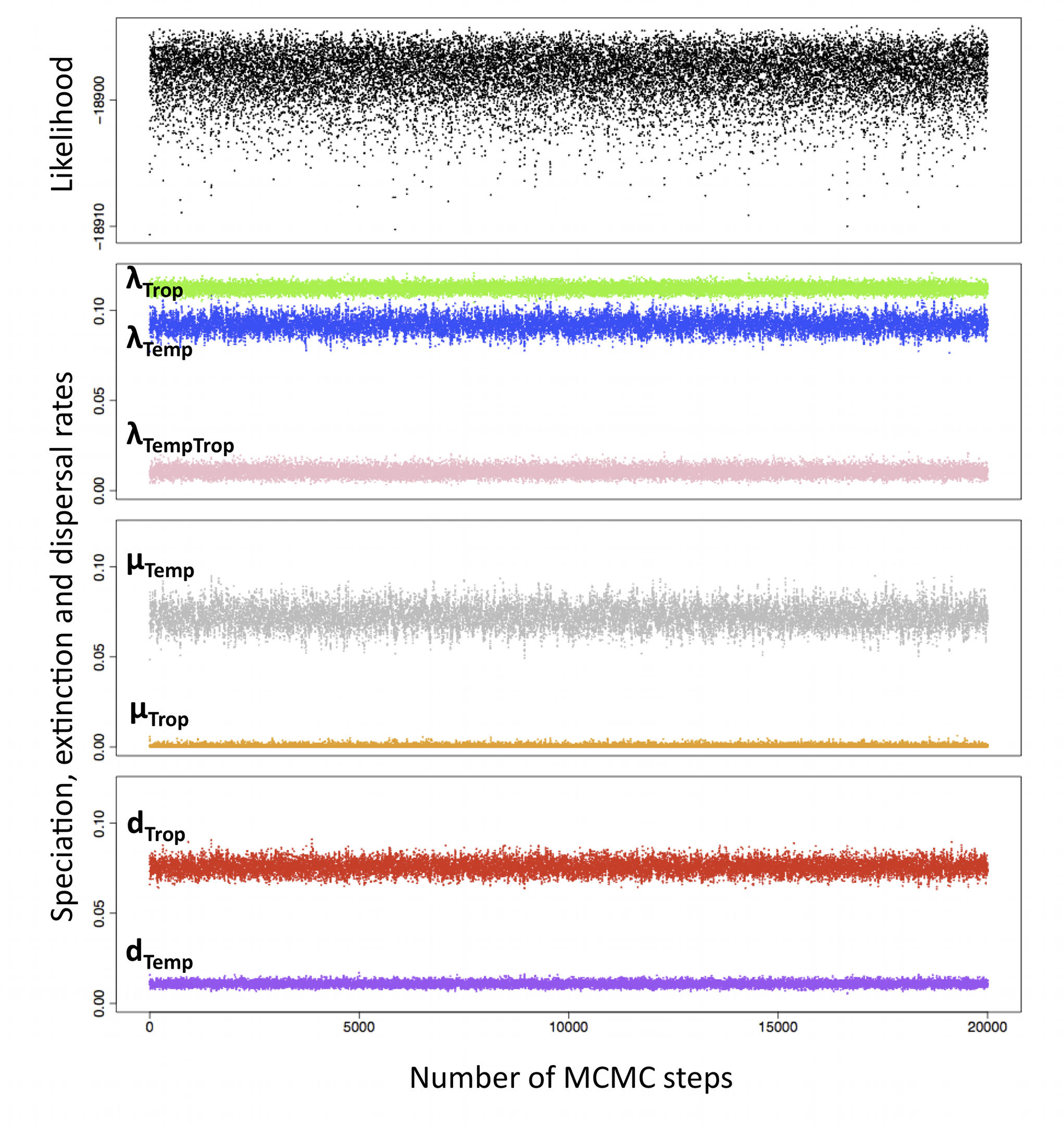

Supplement: Figure S4 — Stability of speciation, extinction, and dispersal estimates along the MCMC. Estimates of speciation and extinction rates in the temperate and tropical biomes along the 20,000 steps of the MCMC following the 500 steps of burnin (not shown in the figure). Parameter estimates are stable along the chain. (TIF) [file pbio.1001775.s005.tif]

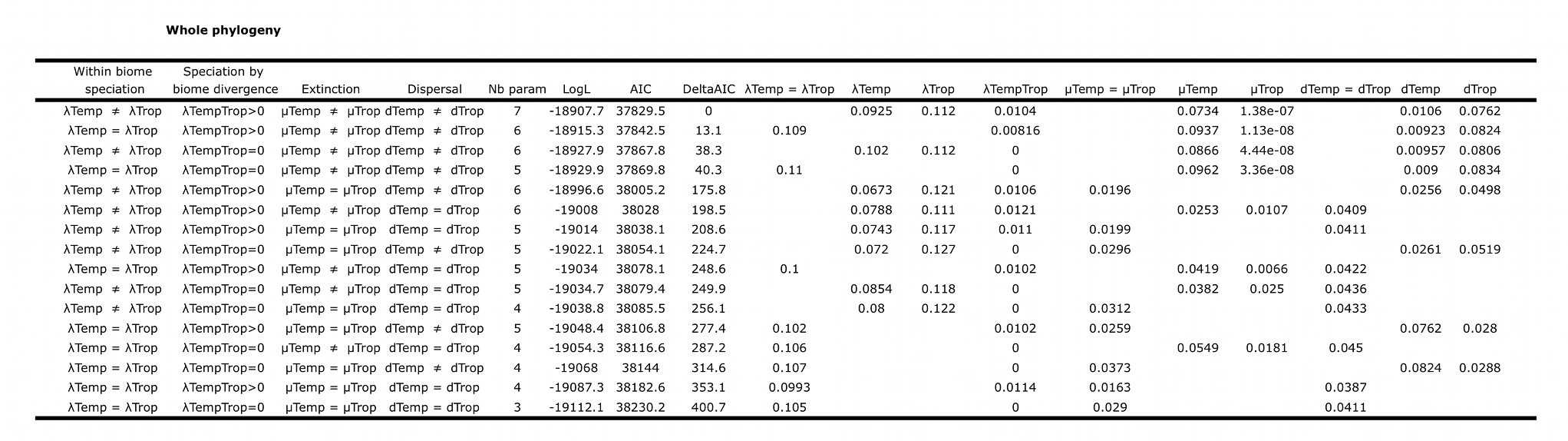

Supplement: Table S1 — Comparison of models for the global, consensus phylogeny. The following table reports results corresponding to the 16 models considered in the article, ranked from best (top) to worst (bottom) fit. Numbers report parameter estimates on the consensus tree. (TIF) [file pbio.1001775.s006.tif]

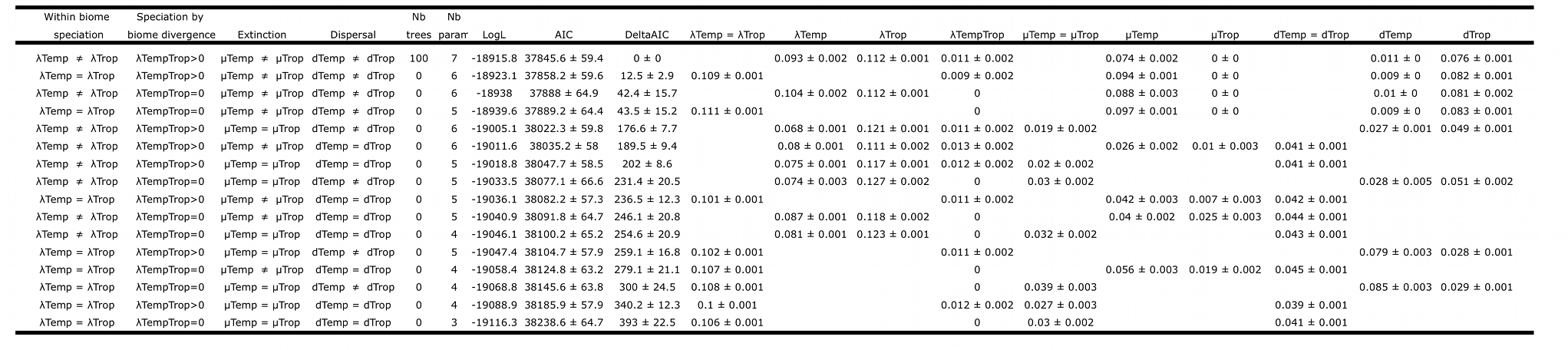

Supplement: Table S2 — Comparison of models for the global phylogeny, using the 100-tree posterior distribution. The following table reports results corresponding to the 16 models considered in the article, ranked from best (top) to worst (bottom) fit. Numbers report parameter and standard deviation estimates from a posterior distribution of 100 trees. Uncertainties between the 100 trees from the posterior distribution and within trees, given by the MCMC analyses for one tree (e.g., Figure 1), were of the same order of magnitude of ∼1×10^−3^ Myr−1 for both unconstrained and constrained analyses. (TIF) [file pbio.1001775.s007.tif]

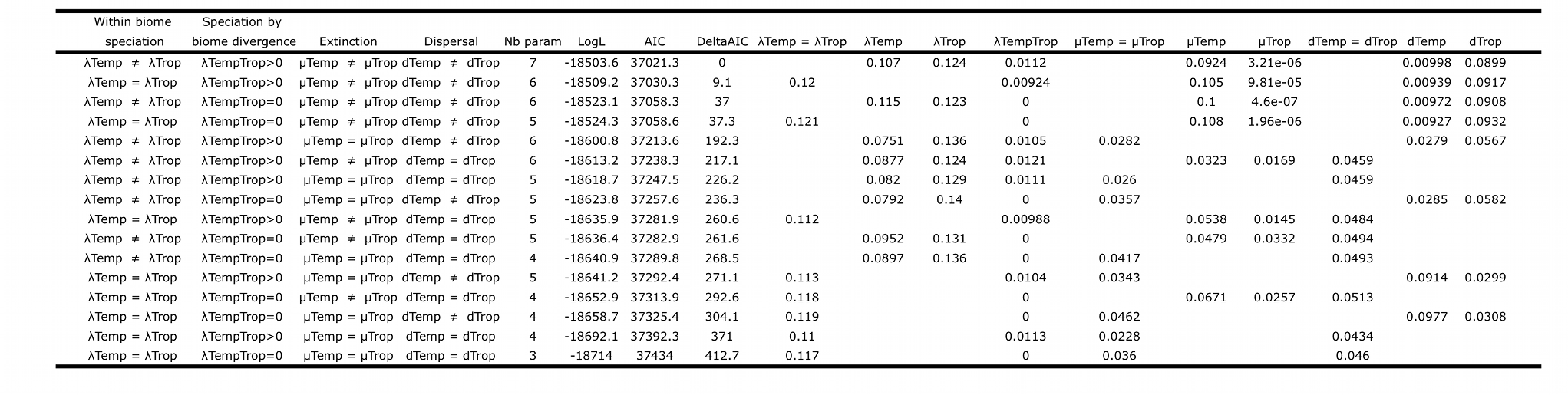

Supplement: Table S3 — Comparison of models for Bininda-Emonds et al.'s [43] tree redated according to Meredith et al. [49] . Results for the eight models considered in the article, ranked from best (top) to worst (bottom) fit. The best-fit model is the model with higher speciation, lower extinction, and higher dispersal rates in the tropics, in agreement with results obtained with the alternative dating. Diversification rate estimates are very similar. (TIF) [file pbio.1001775.s008.tif]

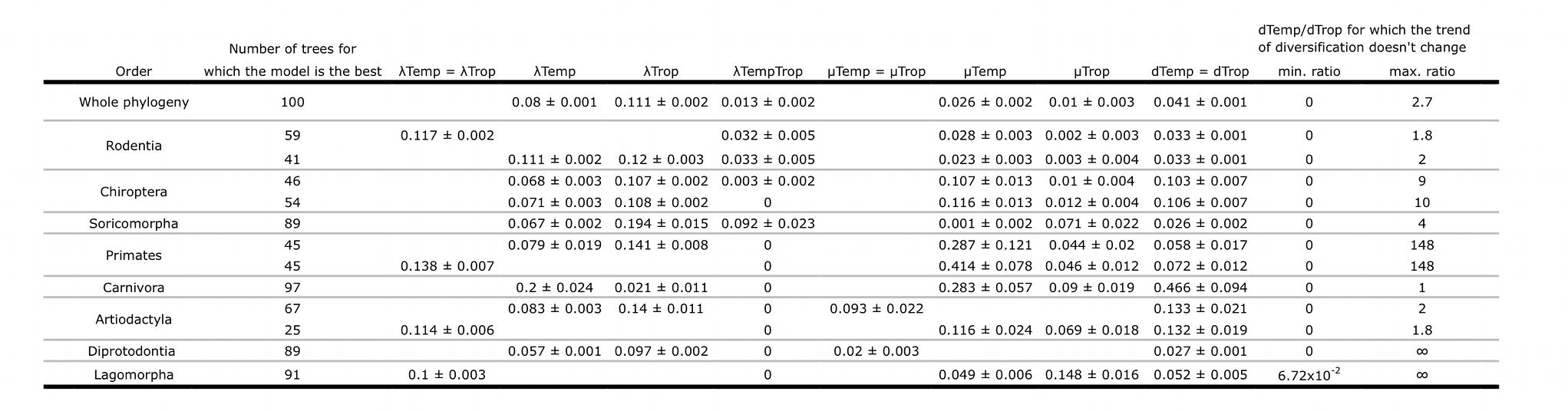

Supplement: Table S4 — Model selection, parameter estimates, and robustness of the results. The second column indicates the number of trees, out of 100, for which the dispersal-constraint model specified in the corresponding row is the best. Models supported by less than 10 trees are not shown. Cells filled with parameter estimates define the model; for example, if parameter estimates are specified in the column headed “λTemp = λTrop,” this indicates that the best-fitting model is a model with equal tropical and temperate speciation rates. The middle columns report mean ± sd parameter estimates over the 100 trees. The two last columns display min and max values of the ratio dTemp/dTrop for which the trend in net diversification rates is conserved. Results were robust to the assumption that range expansion is as frequent from the tropics to the temperate region than in the other direction (dTemp = dTrop). Estimated net diversification rate remains higher in the tropics than in temperate regions for all groups (except the Lagomorpha for which it remained lower) when range expansion is assumed to be less frequent from the temperate to the tropical regions than in the other direction (dTemp/dTrop<1), or when range expansion is reasonably more frequent from the temperate to the tropical regions than in the other direction: 1<dTemp/dTrop<2.7 for the whole phylogeny and 1<dTemp/dTrop<1.8 for orders other than Carnivora. (TIF) [file pbio.1001775.s009.tif]
